# Use of comprehensive genomic profiling for biomarker discovery for the management of non-small cell lung cancer brain metastases

**DOI:** 10.3389/fonc.2023.1214126

**Published:** 2023-11-07

**Authors:** Mohammed Abdulhaleem, John C. Hunting, Yuezhu Wang, Margaret R. Smith, Ralph D’ jr. Agostino, Thomas Lycan, Michael K. Farris, James Ververs, Hui-Wen Lo, Kounosuke Watabe, Umit Topaloglu, Wencheng Li, Christopher Whitlow, Jing Su, Ge Wang, Michael D. Chan, Fei Xing, Jimmy Ruiz

**Affiliations:** ^1^ Department of Internal Medicine (Hematology & Oncology), Wake Forest School of Medicine, Winston-Salem, NC, United States; ^2^ Department of Cancer Biology, Wake Forest School of Medicine, Winston-Salem, NC, United States; ^3^ Department of Biostatistics and Data Science, Wake Forest School of Medicine, Winston-Salem, NC, United States; ^4^ Department of Radiation Oncology, Wake Forest School of Medicine, Winston-Salem, NC, United States; ^5^ Department of Pathology, Wake Forest School of Medicine, Winston-Salem, NC, United States; ^6^ Department of Radiology, Wake Forest School of Medicine, Winston-Salem, NC, United States; ^7^ Department of Biostatistics and Health Data Science, Indiana University School of Medicine, Indianapolis, IN, United States; ^8^ Department of Biomedical Engineering, Rensselaer Polytechnic Institute, Troy, NY, United States

**Keywords:** brain metastasis, non-small cell lung cancer, outcomes, biomarkers, genomic profiling

## Abstract

**Background:**

Clinical biomarkers for brain metastases remain elusive. Increased availability of genomic profiling has brought discovery of these biomarkers to the forefront of research interests.

**Method:**

In this single institution retrospective series, 130 patients presenting with brain metastasis secondary to Non-Small Cell Lung Cancer (NSCLC) underwent comprehensive genomic profiling conducted using next generation circulating tumor deoxyribonucleic acid (DNA) (Guardant Health, Redwood City, CA). A total of 77 genetic mutation identified and correlated with nine clinical outcomes using appropriate statistical tests (general linear models, Mantel-Haenzel Chi Square test, and Cox proportional hazard regression models). For each outcome, a genetic signature composite score was created by summing the total genes wherein genes predictive of a clinically unfavorable outcome assigned a positive score, and genes with favorable clinical outcome assigned negative score.

**Results:**

Seventy-two genes appeared in at least one gene signature including: 14 genes had only unfavorable associations, 36 genes had only favorable associations, and 22 genes had mixed effects. Statistically significant associated signatures were found for the clinical endpoints of brain metastasis velocity, time to distant brain failure, lowest radiosurgery dose, extent of extracranial metastatic disease, concurrent diagnosis of brain metastasis and NSCLC, number of brain metastases at diagnosis as well as distant brain failure. Some genes were solely associated with multiple favorable or unfavorable outcomes.

**Conclusion:**

Genetic signatures were derived that showed strong associations with different clinical outcomes in NSCLC brain metastases patients. While these data remain to be validated, they may have prognostic and/or therapeutic impact in the future.

**Statement of translation relevance:**

Using Liquid biopsy in NSCLC brain metastases patients, the genetic signatures identified in this series are associated with multiple clinical outcomes particularly these ones that lead to early or more numerous metastases. These findings can be reverse-translated in laboratory studies to determine if they are part of the genetic pathway leading to brain metastasis formation.

## Highlights

*Among NSCLC brain metastases, seventy-two genes significantly signaled clinical outcomes.

*Unfavorable outcomes only associated with 14 genes while 36 only favorable.

*Genetic signals strongly indicate the ability to predict oligometastatic disease.

## Introduction

Nearly 170,000 patients a year in the United States are diagnosed with brain metastases (1), and approximately half of brain metastases derive from NSCLC (2). However, even amongst patients with metastases originating from the same primary cancer type, there is a significant diversity of biological and clinical outcomes. A heterogeneity in clinical outcomes derives from a variability in the size and number of metastases (3), along with differences in histology (4) and health status (5, 6) of each patient. The sensitivity of each patient’s cancer to systemic therapy has also been found to be an important variable that contributes to clinical differences between patients (7).

The diversity of the brain metastasis population has made biomarker discovery an important goal. Recent evidence suggests that there may be brain metastasis-specific mutations, and that the genetics of brain metastases may evolve differently from a patient’s primary cancer (8). Several attempts have been made to link genetic signatures found in resected brain metastasis samples to clinical outcomes of brain metastases (9, 10). However, thus far, clinically useful predictive biomarkers for brain metastasis outcomes have been generally elusive.

Over the past several years, several commercial platforms have developed for comprehensive genomic profiling of non-small cell lung cancers (11). Methods for such profiling include genetic sequencing of a biopsied tumor sample, as well as sequencing of circulating tumor DNA (12). Circulating tumor DNA has been shown to correlate with tumor mutations both in serum and cerebrospinal fluid (CSF) (13). Sensitivities have varied, among populations, notably with circulating tumor DNA of the CSF having a higher correlation to brain metastases mutations given the blood brain barrier (13–15). Though plasma samples remain valid and are more easily and commonly obtained prior to known diagnosis of brain metastases. These platforms for genomic profiling of lung cancers has helped to identify potential therapeutic targets in patients that are found to have targetable mutations (16–18), or identify populations that are more likely to respond to immunotherapy (16, 19).

Amongst the brain metastasis population, several potential clinical dilemmas exist for which biomarker discovery could potentially change practice. The ability to predict such outcomes as brain metastasis velocity (20), leptomeningeal disease (21), and which patients benefit from whole brain radiation (22) would give practitioners guidance to select proper therapies for each individual. The goal of the present study is to demonstrate the feasibility of using comprehensive genomic profiling to discover biomarkers that predict brain metastasis outcomes in patients with NSCLC. To this end, we used a single institution retrospective review of NSCLC brain metastasis patients receiving stereotactic radiosurgery (SRS) who underwent comprehensive genomic profiling and analyzed whether the genes assessed in genomic profiling were associated with clinical characteristics and patient outcomes.

## Methods

### Data acquisition, inclusion, and exclusion

The present study was approved by the institutional review board at Wake Forest School of Medicine. Patients were eligible for this study if they had a diagnosis of brain metastasis from NSCLC and had comprehensive genomic profiling performed with at least one mutation detected. Patients without detectable mutations, whether uninformative test with no ctDNA detected or negative test with no alterations found were excluded. The Wake Forest brain metastasis database which prospectively includes all patients receiving stereotactic radiosurgery for brain metastases was searched for all patients with NSCLC. Clinical characteristics and outcomes were determined via the electronic medical records. Tumor and dosing characteristics were determined using the GammaPlan Treatment Planning System (Elekta AB, Stockholm).

A total of 130 patients who had initial diagnosis of brain metastasis between August 2012 - September 2021 were included in the study. Data collected included age, race, gender, smoking status, number of metastases at initial gamma knife (GK), lowest dose prescribed to a metastasis at SRS, Karnofsky performance scale (KPS), systemic disease burden, time of brain metastasis, number of metastases at first distant brain failure, and time of death. Patient characteristics are summarized in **Table 1**.

**Table 1:** Patient Characteristics.

Total = 130 patients	Median (range).
Primary diagnosis (Adenocarcinoma)	97 (75%)
Gender (Female)	68 (52%)
Age (years)	68 (41–85)
Race (White)	108 (83%)
KPS	78 (60–90)
Smoking status	
Former	83 (64%)
Current	26 (20%)
Never	21 (16%)
Lowest GK	18 (12–24)
Number of metastases at first GK	4 (1–18)
Systemic disease burden (oligometastatic)	51 (39%)
Brain metastasis velocity (n 54/130)	14.2 (0 – 210)
Low (≤4)	21/54
Intermediate (4–13)	21/54
High (>13)	12/54
Number of metastases at distant brain failure (n 54/130)	4 (1–35)
Concurrent diagnosis	88 (68%)
Timing relationship between sequencing and GK (days)	
Patients sequenced first later treated with GK (n 67)	25 (12.5 - 103)
Patients who received sequencing after GK (n 63)	188 (22.5 - 384.5)
Frequency of mutated genes (N=77)	
*TP53*	70%
*KRAS*	32%
*EGFR*	26%
*ARID1A, ERBB2, NF1, KTI, STK11, PIK3CA, PDGFRA, AR, MET, BRAF(V600), BRACA1, APC.*	10% - 20%
Other genes	< 10%

Categorical variables reported as count (frequency).

Continuous variables reported as median (interquartile range).

### Stereotactic radiosurgery

Patients included in the study were treated with SRS using the GK Perfexion (Elekta AB, Stockholm, Sweden). Patients were immobilized using rigid frame fixation and underwent a same day high resolution stereotactic magnetic resonance imaging (MRI) with contrast (GE Healthcare, Chicago, IL). Treatment planning was performed using the GammaPlan Treatment Planning System. Dose prescription was done per guidelines derived from the RTOG 90-05 study (23).

### Comprehensive genomic profiling

Comprehensive genomic profiling was conducted using next generation circulating tumor DNA(Guardant Health, Redwood City, CA). Blood sample of cell free DNA obtained from the patients prior to receiving systemic therapy using a CLIA-certified Next Generation Sequencing test as described by Leighl et al. (24). Sequencing included collections both before and after initiating GK treatment. Time to genomic sequencing is further described in **Table 1**.

### Response assessment, follow-up and definition of clinical outcomes

Patients generally underwent a follow-up MRI with clinical evaluation 6-8 weeks after SRS and then every 3 months thereafter for the first 2 years after radiosurgery. If the patient did not experience tumor progression to that point, the imaging follow-up was done less frequently at that point (every 4-6 months).

Brain metastasis velocity (BMV) was defined as previously published by Farris et al. (7). In brief, BMV was defined as the cumulative number of new brain metastases since SRS divided by the number of brain metastases. Time to distant brain failure was defined as the time to development of new brain metastases that were not previously treated with SRS (3). Lowest GK dose was defined as the lowest prescribed dose to any brain metastasis at time of SRS. This factor has previously been used as a surrogate for treatment volume as the lowest GK dose is inversely proportional to the dose delivered to metastases in a fairly linear relationship (25). Systemic disease burden was defined as none, oligometastatic or widespread (26). If patients had ≤5 non-brain metastases without diffuse involvement of any one organ, the patient was considered to have oligometastatic disease, while having >5 metastases or diffuse distant organ involvement is considered widespread disease (27). Concurrent “Synchronous metastasis” was defined as the diagnosis of a brain metastasis within a three-month interval of the diagnosis of the primary NSCLC (28).

### Statistics

Descriptive statistics (means/medians, standard deviations, ranges, counts and percent) were calculated for all outcomes of interest. Separate genetic signatures were created for each of the nine outcomes of interest (brain metastasis velocity, concurrent diagnosis of brain metastases and primary cancer, time to distant brain failure, performance status, lowest SRS dose, number of metastases at treatment failure, number of metastases at diagnosis, extent of extracranial metastatic disease, and overall survival). The approach consisted of a four-step process. The first step was to screen the seventy-seven genes across each of the nine outcomes to identify any genes that had a modest association (p<0.1) with the outcome. For the five continuous outcomes (brain metastasis velocity, performance status, lowest SRS dose, number of metastases at treatment failure, and number of metastases at diagnosis), 2-sample t-tests were used, for the two binary outcomes (concurrent diagnosis of brain metastases and primary cancer and extent of extracranial metastatic disease), Fisher’s exact tests were used, for overall survival, Cox proportional hazard’s regression was used, and for time to distant brain failure, a competing risk model was used to identify genes for each signature.

Next, for each outcome, the genes identified were separated into “protective” and “harmful” categories based on the direction of the point estimate of each individually assessed mutation with outcome compared to absence of the mutation. (i.e., if the presence of a gene was associated with worse outcome (shorter survival) it was considered “unfavorable” whereas if the presence of a gene was associated with an improved outcome (longer survival) it was considered “favorable”). Favorable clinical outcomes included lower hazard ratio of death, lower mean brain metastasis velocity, lower mean number of brain metastases at first GK or distant brain failure (DBF), lower mean first dose of GK required, longer time to DBF, higher mean KPS, increased frequency of oligometastatic disease pattern, and lower frequency of concurrent diagnosis. Unfavorable outcomes were considered as the converse. For the third step, each unfavorable gene received a score of +1 and each favorable gene received a score of -1. These scores were then summed across genes for each outcome. Finally, the overall gene score was created by transforming the gene scores into 3–level ordinal variables (one for each outcome) as follows: if the gene score for an outcome was negative it was coded as -1, if the gene score was 0 it was coded as 0, and if it was positive it was coded as +1.

These nine gene scores were then evaluated for their ability to predict each of the nine outcomes, respectively. For each of the five continuous outcomes, a general linear model was fit with the gene score included as a class variable. Mean values for each of the 3 levels of the gene score were then compared. For the two binary outcomes, Chi-square tests were performed to assess the gene score. For the time to event outcomes, Cox proportional hazard’s regression models were fit and hazard ratios were examined based on the gene scores. For evaluating the success of the gene scores (signatures), p<0.05 was used to identify significant scores. Multiple testing corrections was not performed.

## Results

### Identification of genes for inclusion in gene signatures for profiling brain metastasis related outcomes

**Table 2** summarizes the genes identified that had a statistically significant association for each clinical outcome of interest. As can be seen, the number of genes included in each signature ranged from six (for Overall Survival) to twenty-eight (for Brain Metastasis Velocity). Across the different outcomes, more genes were identified as favorable than unfavorable, meaning that for several outcomes, having a gene present was associated with not having the negative outcome (i.e., the presence of the *ATM* gene mutation was associated with a more favorable Brain Metastasis Velocity, fewer metastases at first GK, fewer metastases at DBF and the presence of an oligometastatic disease burden).

**Table 2:** Clinical outcomes and their associated genes signature.

Clinical outcomes	Genes Predictive of Favorable Outcome		Genes Predictive of Unfavorable Outcome	
	P-value		P-value	
Overall survival	*KRAS* *CDK6*	*.094* *.092*	*NRAS* *RIT1* *RAF1* *ALK_EML4*	*0.128* *0.173* *0.551* *0.10*
BMV	*AKT1* *APC* *AR* *ATM* *CCND1* *CCND2* *CDH1* *CDK6* *CDK12* *CDKN2A* *CTNNB1* *EGFR* *ERBB2* *FGFR1* *GATA3* *HFN1A* *MAP2K2* *MTOR* *NOTCH1* *NTRK3* *PALB2* *SMAD4*	*.0065* *.0263* *.0391* *.0175* *.0046* *.0049* *.0838* *.0778* *.0043* *.0046* *.0171* *.0974* *.0497* *.0197* *.0046* *.0041* *.0507* *.0069* *.0297* *.0046* *.0163* *.0228*	*CDK4* *FGFR2* *KIT* *MYC* *NFE2l2* *PDGFRA*	*.0408* *.0181* *.0925* *.0119* *.0006* *.0442*
Number of metastases at first GK	*APC* *ATM* *BRCA1* *CCND2* *FGFR1* *FGFR3* *FGFR3_TACC3* *GATA3* *HRAS* *MET* *NOTCH1* *NTRK3* *RAF1* *TSC1*	*.0961* *.0527* *.0002* *<.0001* *.0017* *.0373* *.0413* *<.0001* *.0022* *.0720* *.0049* *.0402* *.0003* *.0022*	*BRAF* *EGFR* *NRAS* *PDGFRA* *RAD51D* *RB1*	*.0741* *.0830* *.0423* *.0516* *.0089* *.0815*
Lowest GK dose	*ALK*, *EML4_ALK* *FBXW7* *HRAS* *IDH2* *SMO* *STK11* *TCI1*	*.048* *.0696* *<0.001* *<0.001* *.0696* *<0.001* *.0028* *.002*	*BRCA2* *CCND1* *EGFR* *MAP2K2* *NF1* *NRAS* *RHEB* *SMAD4*	*.02* *.013* *.097* *.018* *.0258* *.0217* *.0109* *.009*
Number of metastases at DBF*	*AKT1* *ATM* *CCND1* *CCND2* *CDH1* *CDK6* *CDK12* *CDKN2A* *CHEK2* *CTNNB1* *FGFR2* *GATA3* *HFN1A* *MET* *MTOR* *NOTCH1* *NTRK3* *PALB2* *SMAD4* *SMO*	*.0013* *.0115* *.0015* *.0015* *.0229* *.0572* *.0015* *.0015* *.0095* *.0657* *.0583* *.0013* *.0013* *.0927* *.0012* *.0657* *.0013* *.0650* *.0062* *.0095*	*KIT* *PDGFRA*	*.008* *.513*
Time for distant brain failure.	*NRAS* *RET* *FGFR3_TACC3* *EML4_ALK* *FBXW7* *KEAP1* *TSC1* *EZH2* *GNA11* *LRIG3_ROS1* *MAP2K1* *ALK_EML4* *RIT1* *FANCA* *ARAF* *NTRK2* *RHEB*	*<.0001* *<.0001* *<.0001* *<.0001* *<.0001* *<.0001* *<.0001* *<.0001* *<.0001* *<.0001* *<.0001* *<.0001* *<.0001* *<.0001* *<.0001* *<.0001* *<.0001*	*PMS2*, *IDH1*, *MAP2K2*, *GNAQ*, *HRAS*, *APC*	*<.001* *<.0001* *.0004* *.0006* *.0032* *.0495*
KPS	*PMS2*	*.0163*	*AKT1* *CDH1* *FBXW7* *GATA3* *GNAS* *HFN1A* *HRAS* *MAP2K2* *MAPK1* *NTRK3* *SMO*	*.0342* *.0342* *.0342* *.0342* *.0342* *.0342* *.0342* *.0342* *.0342* *.0869* *.0342*
Disease burden (Oligometastatic)	*ATM* *JAK2* *MAP2K2* *NTRK1*	*.048* *.058* *.058* *.058*	*ARID1A* *CCNE1*	*.097* *.088*
Concurrent diagnosis (synchronous)	*MYC*, *NTRK1* *PTEN* *RB1* *AKT1* *GATA3*	*.057* *.032* *.036* *.085* *.099* *.099*	*FGFR2*	*.031*

*BMV, Brain Metastasis Velocity; DBF, Distant Brain Failure.

As described in the methods above, each patient included in the analysis was given a gene signature for each outcome. These scores took on one of three potential values. Genes predictive of a favorable or unfavorable outcome for each clinical endpoint were assigned a value of -1 or -+1, respectively, and then scores were summed to determine risk profile for each patient. Patients with positive sum were considered unfavorable risk, those with a negative sum were considered favorable risk, and those with zero sum were neutral. For example, for the outcome of overall survival there were 6 genes included in the signature, 2 favorable (*KRAS* and *CDK6*) and 4 unfavorable (*NRAS, RIT1, RAF1 or ALK_EML4*). If a patient had one or both favorable genes (*KRAS* and *CDK4*) and no unfavorable genes, then they would be assigned a -1 and considered to have a favorable gene signature. Likewise, if a patient had no favorable genes, but had at least one or more of the unfavorable genes (*NRAS, RIT1, RAF1 or ALK_EML4*) they would be assigned a +1 for their overall survival gene signature. Finally, if a patient had either an equal number of favorable and unfavorable gene mutations, or had no favorable or unfavorable genes present, then they would be assigned a 0 for their overall survival gene score.

**Figure 1:**
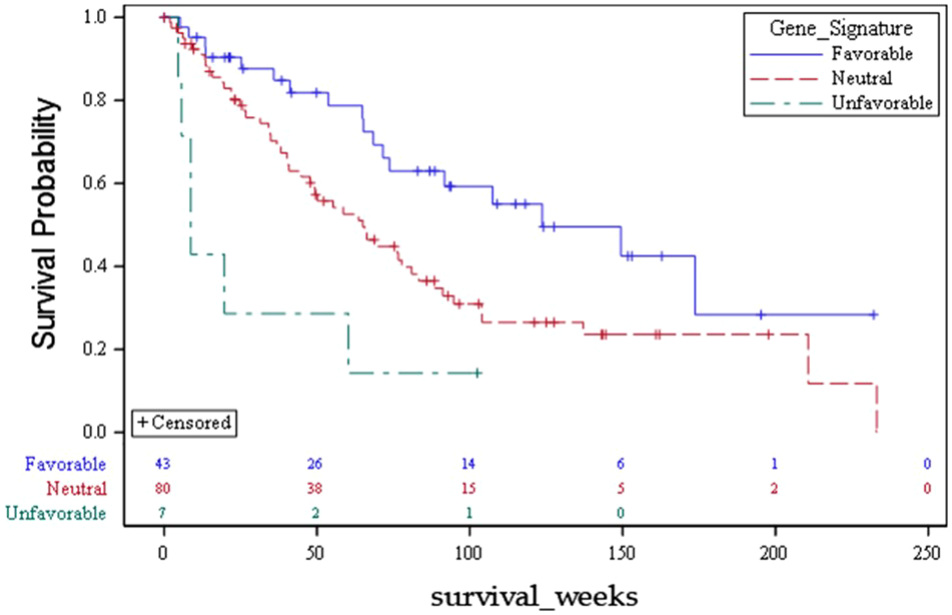
Kaplan Meier Plot for genetic signatures associated with Overall Survival.

**Figure 2:**
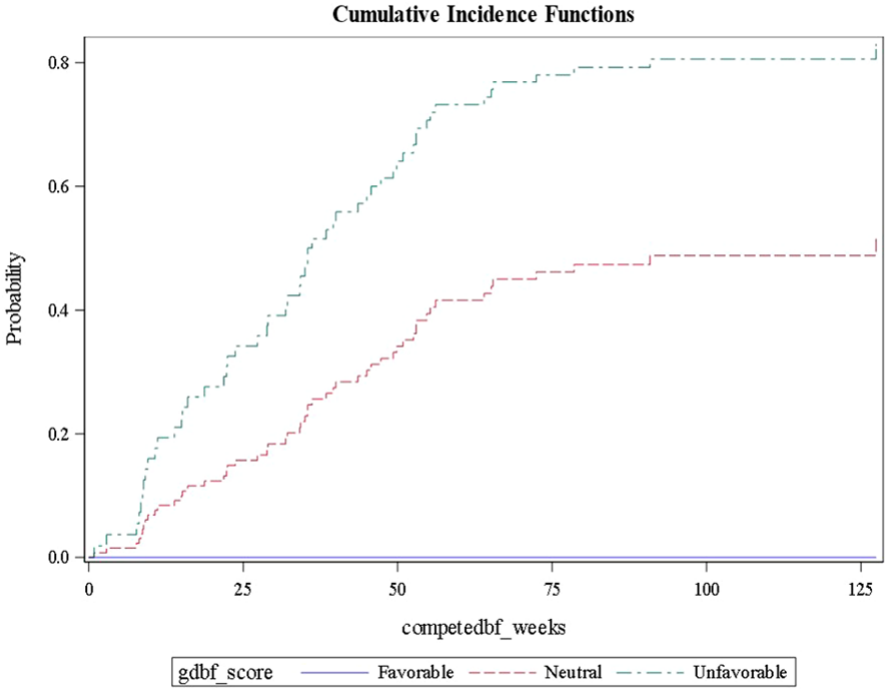
Cumulative Incidence Plot for genetic signatures associated with Distant Brain Failure.

**Figure 3:**
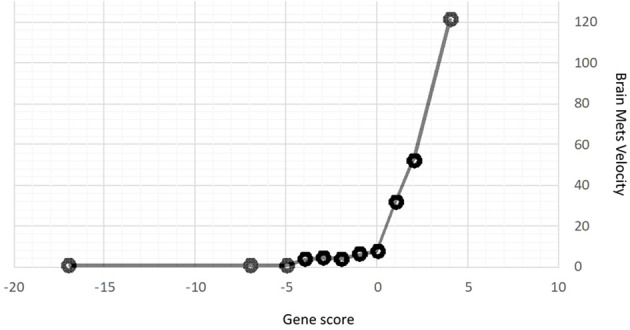
Brain Metastasis Velocity by Gene Signature Score.

Using this approach for each outcome, **Table 3** shows the comparisons of results for each outcome stratified by gene signature. As can be seen, all nine gene signatures were statistically significant for predicting the outcome of interest. In fact, most gene signatures showed a highly statistically and clinically significant difference across values of the signature. **Figures 1**–**3** also display graphically the ability of the gene signatures to differentiate patients for the outcomes of Brain Metastasis Velocity, overall survival and time to distant brain failure (accounting for the competing risk of death).

**Table 3:** Statical significance of genetic signature in association with the clinical outcomes.

	Favorable*	Neutral**	Unfavorable***	P Value
BMV (mean)	4.75	7.85	51.1	0.0002
Lowest Dose at GK (mean) with corresponding brain metastasis volume	20.7 Gy (1 cc)	19.2 Gy (2 cc)	17.5 Gy (6 cc)	<0.0001
Number Metastases at 1^st^ GK (mean)	2.3	3.7	7.2	<0.0001
Number Brain Metastases at DBF (mean)	1.2	3.2	9.2	0.0007
KPS (mean)	80.4	78.3	60	0.026
Oligometastatic extracranial disease (%Yes)	78%	41%	11.5%	<0.0001
Concurrent Diagnosis of Brain Metastasis and Extracranial Disease (%Yes)	22.7%	75.5%	100%	<0.0001
DBF (% Yes)	0%	43%	83%	<0.0001
OS (median in weeks)	124	65	9	0.0017

*Sum of gene values with predictive ability is a positive integer.

**Sum of gene values with predictive ability is zero.

***Sum of gene values with predictive ability is a negative integer.

It should be noted that Karnofsky performance status did not have as strong a statistically significant genomic signature association as other endpoint signatures. The difference between neutral and favorable signatures was KPS of 78 *vs* 80, respectively, and the unfavorable signature only represented one patient with a KPS of 60.

### Genes associated with multiple outcomes

Several genes were associated with multiple clinical outcomes. These are summarized in **Table 4**. Genes associated with improved clinical outcomes included *AKT1* (greater likelihood of oligometastatic disease, lower number of metastases at first SRS and distant brain failure), *CDK6* (lower BMV and lower number of metastases at distant brain failure), and *GATA3* (better KPS, lower BMV, lower number of metastases at first GK and distant brain failure). Genes associated with a multiple negative clinical outcome include: *NRAS* (lower dose delivered at SRS and greater number of metastases at first SRS), and *PDGFRA* (higher BMV, greater number of metastases at first SRS and at distant brain failure).

**Table 4:** Single genes and associated with multiple clinical outcomes.

**Genes**	**Favorable clinical** **outcomes**	**Unfavorable clinical outcomes**
*ATK1*	Oligometastatic disease, lower number of metastases at first GK and DBF,	NA
*CDK6*	Lower BMV, lower number of metastases at DBF	NA
*GATA3*	Lower KPS, lower number of metastases at first GK and DBF	NA
*NRAS*	NA	Higher SRS dose, and number of metastases at first GK
*PDGFRA*	NA	Higher number of metastases at first GK and DBF, and higher BMV

Table notation for associated clinical outcomes with genes identified of interest.

NA (not-applicable) listed for outcomes when the association is understood to be the converse.

## Discussion

NSCLC represents a genetically diverse population. In the late 1990’s, subpopulations of NSCLC patients who were found to be predominantly female non-smokers were identified and found to have cancers that responded to tyrosine kinase inhibitors (29). The mechanism of this response has been determined to be an activating mutation in the *EGFR* gene (30). This discovery led to a cascade of subsequent discoveries of various subpopulations of the NSCLC population including the ALK (31), ROS-1 (32), RET (33) and BRAF-mutated (34) populations. While the aforementioned mutations represent activating mutations for which targeted agents have been developed to counter, these may not represent the full story for how genomic analysis may ultimately affect care in NSCLC patients.

Biomarker discovery for brain metastasis behavior has thus far been an elusive process (35). This difficulty derives from several reasons including the histologic heterogeneity of the brain metastasis population (3), and the propensity for continued mutation between a primary tumor and the clonogens that ultimately become brain metastases (8). A preliminary study found that assessing circulating DNA in the serum as done in the present study may better detect mutations not found in a primary colorectal tumor though these findings would need to be validated in lung cancer patients (36). The present study also attempted to address the issue of histologic heterogeneity by using a population of purely NSCLC patients. Future investigations will likely include attempts to use tissue acquired from craniotomy samples in order to ensure that mutations from brain metastases are captured in the genomic analyses (9).

In the present analysis, genetic signatures were discovered for factors that have the potential to affect management. For example, patients with a signature predicting a lower SRS dose represent a population in which a dominant brain metastasis developed that was generally large and/or symptomatic. These brain metastases are ones that historically lead to significant morbidity and mortality (37, 38). Such a signature could yield a population for which surveillance imaging even prior to brain metastasis diagnosis may be useful. In addition, patients with signatures for lower BMV or lower number of brain metastases at distant brain failure may ultimately represent populations for which aggressive use of SRS is justified (39, 40), perhaps even in cases when a greater number of metastases are present than are normally offered SRS (41).

Clinical outcomes in the present analysis were scored with regards to whether they were favorable or unfavorable in the clinical setting. For example, larger or more numerous brain metastases or higher BMV were considered adverse, whereas smaller or fewer brain metastases were considered protective. This allowed for assessment of whether single genes could be favorable or unfavorable in multiple outcomes. Several gene mutations were identified within the multiple genetic signatures and these genes were found to be more likely to be either favorable or unfavorable (as opposed to discordant) across those multiple outcomes. Ultimately, genetic mutations identified in multiple unfavorable signatures are candidates, particularly in outcomes that lead to early or more numerous metastases, to be reverse-translated in laboratory studies to determine if they are part of the genetic pathway leading to brain metastasis formation.

The meaningful clinical separation between risk strata determined in the present series was quite large. For example, the survival difference between favorable, neutral, and unfavorable strata was 124 weeks, 65 weeks, and 9 weeks respectively (p=0.002). Patients with life expectancy as low as 2 months of survival may choose to have treatments that can be costly and affect quality of life. Moreover, the corresponding tumor volumes predicted for each risk strata by the present analysis based on lowest GK dose are 1 cc (favorable), 2 cc (neutral) and 6 cc (unfavorable). Tumors of the favorable or neutral scores tend to be good candidates for SRS whereas those with unfavorable scores had tumor volumes that are often best managed with surgery. These findings with regards to presenting tumor volume suggest that there may be volumetric phenotypes for brain metastasis presentation (large symptomatic *vs* small asymptomatic) which are driven by biology. Such a hypothesis has significant reverse translational potential.

That KPS was the single clinical characteristic assessed for which the identified signature demonstrated a weaker statistical association and minimal clinical impact served to strengthen the argument that the other signatures may be valid. Other clinical factors such as BMV, number of brain metastases and size of brain metastases at presentation are factors for which there is a reasonable assumption of a biological phenotype responsible for the size and rate of seeding of the brain with cancer. KPS on the other hand is a complex variable dependent upon multiple factors such as age, burden of systemic disease, comorbidities, and ability to access care (42, 43). Many of these factors are beyond the scope of the genetics of a patient’s cancer, and thus it would not be expected that a genetic signature could be found for KPS, but it was significant finding that this was the one variable that could not be predicted by a genomic signature in our dataset, functioning essentially as a negative control.

One of the genetic associations found in the present series was the signature for having oligometastatic extracranial disease. It has been hypothesized that a certain subset of cancers are truly oligometastatic, and thus, limited in their burden of tumor spread (44). As such, these cancers may benefit from local therapies directed towards the few sites of disease. There have been several recently published series that have suggested that such local treatment of extracranial oligometastatic disease can be beneficial for patients with regards to endpoints such as progression free survival, overall survival and need for systemic therapy (45). However, while some patients benefit and truly have a limited burden of metastatic disease, there are others that will experience rapid and diffuse failure, essentially rendering the local therapies non-useful. As with other relevant signatures found in the present series, the true clinical spread between favorable and unfavorable was quite large, as patients with favorable predictive score for oligometastatic disease had a 78% likelihood of having true oligometastatic disease. Conversely, those with unfavorable predictive score only had a 12% risk of having oligometastatic disease. If these genomic signatures for oligometastatic disease are validated, then they will have the potential to help dictate which patients may be candidates for local extracranial therapies.

There are several limitations to the present study. The study is a retrospective analysis and is therefore subject to selection bias. There also exists the issue of circulating DNA sampling as cancers are known to continue to mutate and the mutations found in the circulating DNA may or may not be present in the brain metastases. Additionally, only half the population had ctDNA drawn prior to SRS adding the potential for confounding. Circulating DNA from the CSF has been shown to correlate more closely to mutations of CNS involvement such as leptomeningeal disease (46) and brain metastases, though remain a more invasive procedure via lumbar puncture and less standardized than blood draw. If validated, however, the identified genomic signatures in this series represent clinically useful data obtained via non-invasive liquid biopsy to help risk stratify patients to potentially inform treatment or surveillance decisions.

## Data availability statement

The data analyzed in this study is subject to the following licenses/restrictions: Data will be made available per request to the corresponding author. Requests to access these datasets should be directed to John Hunting, jhunting@wakehealth.edu.

## Ethics statement

The studies involving humans were approved by Wake Forest School of Medicine IRB. The studies were conducted in accordance with the local legislation and institutional requirements. Written informed consent for participation was not required from the participants or the participants’ legal guardians/next of kin in accordance with the national legislation and institutional requirements.

## Author contributions

MA, MC, FX contributed to conception and design of the study. YW, MA, H-WL, KW, UT procured relevant data. RD’A, JS, GW performed the statistical analysis. MA wrote the first draft of the manuscript. All authors contributed to manuscript revision, read, and approved the submitted version.
